# Current status of laparoscopic bariatric/metabolic surgery in Japan: The sixth nationwide survey by the Japan Consortium of Obesity and Metabolic Surgery

**DOI:** 10.1111/ases.12836

**Published:** 2020-07-21

**Authors:** Masayuki Ohta, Kazunori Kasama, Akira Sasaki, Takeshi Naitoh, Yosuke Seki, Susumu Inamine, Takashi Oshiro, Yuichiro Doki, Yasuyuki Seto, Hideki Hayashi, Ichiro Uyama, Shuji Takiguchi, Kazuyuki Kojima, Toshiyuki Mori, Masafumi Inomata, Yuko Kitagawa, Seigo Kitano, Japan Consortium of Obesity and Metabolic Surgery (JCOMS)

**Affiliations:** ^1^ Global Oita Medical Advanced Research Center for Health Oita University Yufu Japan; ^2^ Weight Loss and Metabolic Surgery Center Yotsuya Medical Cube Tokyo Japan; ^3^ Department of Surgery Iwate Medical University School of Medicine Morioka Japan; ^4^ Department of Lower Gastrointestinal Surgery Kitasato University School of Medicine Sagamihara Japan; ^5^ Department of Surgery Ohama Dai‐ichi Hospital Okinawa Japan; ^6^ Department of Surgery Toho University Sakura Medical Center Sakura Japan; ^7^ Department of Gastroenterological Surgery Osaka University Graduate School of Medicine Osaka Japan; ^8^ Department of Gastrointestinal Surgery University of Tokyo Tokyo Japan; ^9^ Department of Frontier Surgery Chiba University Graduate School of Medicine Chiba Japan; ^10^ Department of Surgery Fujita Health University School of Medicine Toyoake Japan; ^11^ Department of Gastroenterological Surgery Nagoya City University Nagoya Japan; ^12^ First Department of Surgery Dokkyo Medical University Tochigi Japan; ^13^ Department of Surgery Kyorin University School of Medicine Mitaka Japan; ^14^ Department of Gastroenterological and Pediatric Surgery Oita University Faculty of Medicine Oita Japan; ^15^ Department of Surgery Keio University School of Medicine Tokyo Japan; ^16^ Oita University Oita Japan

**Keywords:** bariatric surgery, laparoscopic bariatric surgery, metabolic surgery

## Abstract

**Introduction:**

Laparoscopic bariatric procedures have been performed in Japan since 2000. Laparoscopic sleeve gastrectomy (LSG) has been fully covered by National Health Insurance since 2014, and it has been increasingly performed recently. The Japan Consortium of Obesity and Metabolic Surgery conducts a nationwide survey on laparoscopic bariatric/metabolic surgery every 2 years.

**Methods:**

The survey was sent by post or email to 97 Japanese institutions in January 2020.

**Results:**

From 2000 to 2019, a total of 3669 laparoscopic bariatric/metabolic procedures were performed in 64 institutions. The most popular procedure was LSG (n = 2866), followed by LSG with duodenojejunal bypass (LSG‐DJB, n = 337) and laparoscopic Roux‐en‐Y gastric bypass (LRYGB, n = 280). Morbidity and reoperation rates were, respectively, 29.8% and 11.8% for LRYGB, 16.8% and 2.8% for LSG, and 13.6% and 6.6% for LSG‐DJB. At 5 years after the procedures, the percentage of excess weight loss was 78% for LRYGB, 66% for LSG, and 80% for LSG‐DJB.

**Conclusion:**

This nationwide survey clearly showed that laparoscopic bariatric/metabolic surgery has been safely and effectively performed for 20 years in Japan.

## 1 INTRODUCTION

In Japan, a group at Chiba University started performing bariatric surgery in 1982, and the National Health Insurance first approved open vertical banded gastroplasty as a gastric restriction procedure in 1988.^1^ However, at that time, only a few institutions performed it. Since 2000, laparoscopic bariatric procedures, including laparoscopic Roux‐en‐Y gastric bypass (LRYGB), laparoscopic adjustable gastric banding (LAGB), and laparoscopic sleeve gastrectomy (LSG), began to be introduced in Japan as the number of morbidly obese patients who were refractory to medical treatments increased.^1^, ^2^ In 2005, National Health Insurance had yet to approve any laparoscopic bariatric procedures, so the Japan Research Society for Endoscopic and Laparoscopic Treatments of Obesity (JELTO), which had been preceded by the Laparoscopic Bariatric Surgery Forum, was established to spread and develop safe endoscopic and laparoscopic bariatric procedures in Japan. This society, which is officially associated with the Japan Society for Endoscopic Surgery (JSES),^3^ conducted its first national survey on laparoscopic bariatric surgery in 2010.^1^ LSG was approved as an advanced medical treatment in 2010, at which time it was partly covered by National Health Insurance; since 2014, it has been fully covered.

In 2014, JELTO was renamed the Japan Consortium of Obesity and Metabolic Surgery (JCOMS) to indicate a focus not only on bariatric surgery but also on metabolic surgery. JCOMS has continued the nationwide survey on laparoscopic bariatric/metabolic surgery started by JELTO in 2010 and conducts it every 2 years. Here, we report the current status of laparoscopic bariatric/metabolic surgery in Japan based on the results of the sixth nationwide survey conducted by JCOMS.

## 2 MATERIALS AND METHODS

In January 2020, a postal survey was mailed to 76 member institutions of JCOMS, and an email survey was sent to 21 non‐member institutions that were known to have performed bariatric/metabolic surgery. The survey included three questions covering data through the end of 2019:

1. Does your institution have experience with laparoscopic bariatric/metabolic surgery?
2. How many cases of each laparoscopic bariatric/metabolic procedure were performed in your institution in 2018 and 2019?
3. What have been the results of the four major bariatric/metabolic procedures (LRYGB, LAGB, LSG, and LSG with duodenojejunal bypass [LSG‐DJB]), including preoperative weight and BMI, complications, rates of reoperation, mean weight loss, and mean percentage of excess weight loss (%EWL)?

Postoperative gastroesophageal reflux disease (GERD) was defined as having GERD symptoms and requiring the administration of a proton‐pump inhibitor (PPI).

If necessary, follow‐up emails were sent to obtain the data requested. Ultimately, 60 of the 76 member institutions (78.9%) replied to the questionnaire, whereas all non‐member institutions (100%) did. This survey did not require any personal information from patients. It was approved by the Ethics Committee of Oita University Faculty of Medicine (no. 1754).

The cumulative number of institutions and cases were calculated by adding the data from the past five surveys. The data from the 2020 survey were calculated for complications, reoperation rate, and weight loss. The tabular data were received, and certain derivative data were calculated. Data regarding preoperative weight and BMI, weight loss, and %EWL for the major procedures were calculated from the mean values and number of operations at each institution. Excess weight was calculated based on actual weight and ideal weight (BMI 25 kg/m^2^), and %EWL was calculated as reported previously.^4^

## 3 RESULTS

### 3.1 Cumulative number of experienced institutions and laparoscopic bariatric/metabolic procedures

**FIGURE 1:**
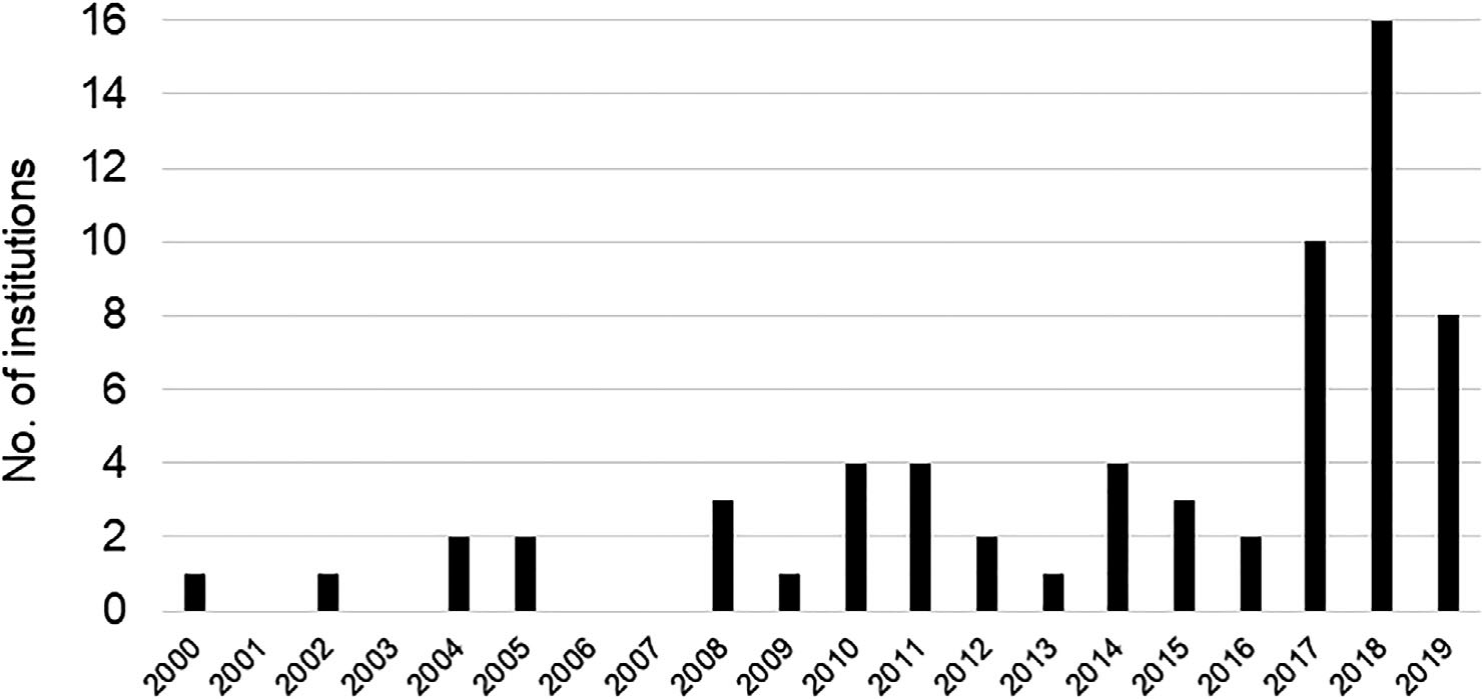
First year in which responding Japanese institutions performed laparoscopic bariatric/metabolic surgery

Laparoscopic bariatric/metabolic surgery was performed at 64 Japanese institutions through the end of 2019. Figure 1 shows the year each institution first performed this type of surgery. One institution first performed hand‐assisted LRYGB in 2000, and another performed completely LRYGB in 2002. The number of institutions that have introduced these procedures has been increasing since 2017. At one institution, more than 1000 laparoscopic bariatric/metabolic surgeries have been performed, and at another eight institutions, more than 100 have been performed at each. Laparoscopic bariatric/metabolic surgery was performed at 59 of the 64 institutions (92.2%) between 2018 and 2019.

**FIGURE 2:**
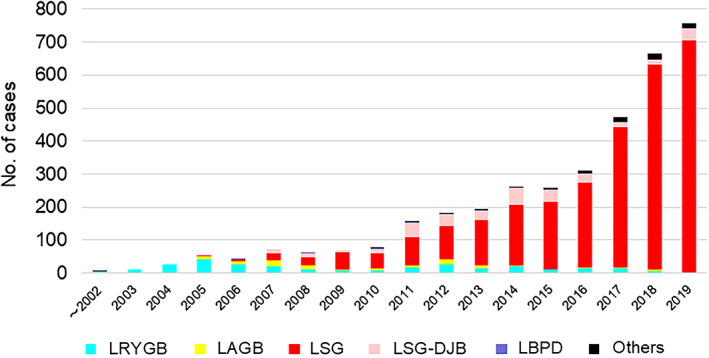
Number of laparoscopic bariatric/metabolic surgeries performed yearly. LAGB, laparoscopic adjustable gastric banding; LBPD, laparoscopic biliopancreatic diversion; LRYGB, laparoscopic Roux‐en‐Y gastric bypass; LSG, laparoscopic sleeve gastrectomy; LSG‐DJB, laparoscopic sleeve gastrectomy with duodenojejunal bypass

In total, 3669 laparoscopic bariatric/metabolic procedures were performed in Japan from 2000 to 2019. The most popular procedure was LSG (n = 2866), followed by LSG‐DJB (n = 337). LRYGB was performed in 280 patients, LAGB in 109, biliopancreatic diversion in 7, and other procedures in 70. Figure 2 shows the number of procedures performed yearly and clearly demonstrates a rapid increase in institutions performing LSG. In 2019, laparoscopic bariatric/metabolic procedures were performed in 757 patients, with 704 (93.0%) undergoing LSG.

### 3.2 Results of the four major bariatric/metabolic procedures

Patient outcomes were analyzed in 3570 of the 3669 cases (97.3%). Total morbidity rates were 29.8% for LRYGB, 11.8% for LAGB, 16.8% for LSG, and 13.6% for LSG‐DJB (Table 1). Comparatively frequent complications (≥2%) involved anastomotic leakage, anastomotic stenosis, and anastomotic ulcer in LRYGB; band slippage and port/tube trouble in LAGB; postoperative GERD in LSG; and postoperative bleeding, gastric tube stenosis, and GERD in LSG‐DJB. Reoperation rates were 11.8% for LRYGB, 16.7% for LAGB, 2.8% for LSG, and 6.6% for LSG‐DJB; in particular, LSG was safely performed. Three patients (0.1%) died postoperatively, one of multiple organ failure due to postoperative hemorrhage and shock after LAGB, another of leakage and propofol infusion syndrome after LSG, and the third of pulmonary failure >30 days after LRYGB.

**TABLE 1:** Complications after the major bariatric/metabolic procedures

	LRYGB (n = 271)	LAGB (n = 102)	LSG (n = 2865)	LSG‐DJB (n = 332)
Total morbidity	81 (29.8%)	12 (11.8%)	482 (16.8%)	45 (13.6%)
Intraoperative complications (open conversion)	1 (0.4%)	0 (0%)	5 (0.2%)	0 (0%)
Postoperative complications				
Bleeding (reoperation needed)	4 (1.5%)	1 (1.0%)	21 (0.7%)	8 (2.4%)
Anastomotic leakage	12 (4.4%)	0 (0%)	14 (0.5%)	3 (0.9%)
Intra‐abdominal abscess	3 (1.1%)	0 (0%)	6 (0.2%)	1 (0.3%)
Anastomotic stenosis	28 (10.3%)	0 (0%)	0 (0%)	0 (0%)
Anastomotic ulcer	20 (7.4%)	0 (0%)	0 (0%)	0 (0%)
Gastric fistula	2 (0.7%)	0 (0%)	0 (0%)	0 (0%)
Internal hernia	4 (1.5%)	0 (0%)	0 (0%)	1 (0.3%)
Band slippage	0 (0%)	2 (2.0%)	0 (0%)	0 (0%)
Port/tube trouble	0 (0%)	9 (8.8%)	0 (0%)	0 (0%)
Gastric tube stenosis	0 (0%)	0 (0%)	32 (1.1%)	7 (2.1%)
Gastroesophageal reflux disease	0 (0%)	0 (0%)	346 (12.1%)	13 (3.9%)
Wound infection	1 (0.4%)	0 (0%)	35 (1.2%)	2 (0.6%)
Subcategory of postoperative complications	6 (2.2%)	0 (0%)	23 (0.8%)	10 (3.0%)
Reoperation rate	32 (11.8%)	17 (16.7%)	79 (2.8%)	22 (6.6%)
Early reoperation (≤30 d)	16 (5.9%)	2 (2.0%)	32 (1.1%)	11 (3.3%)
Late reoperation (>30 d)	16 (5.9%)	15 (14.7%)	47 (1.6%)	11 (3.3%)
Mortality	1 (0.4%)	1 (1.0%)	1 (0.0%)^a^	0 (0%)

*Note*: All figures are presented as frequency (percentage). Abbreviations: LAGB, laparoscopic adjustable gastric banding; LRYGB, laparoscopic Roux‐en‐Y gastric bypass; LSG, laparoscopic sleeve gastrectomy; LSG‐DJB, laparoscopic sleeve gastrectomy with duodenojejunal bypass. ^a^Mortality occurred in 0.03% of patients who underwent LSG.

**FIGURE 3:**
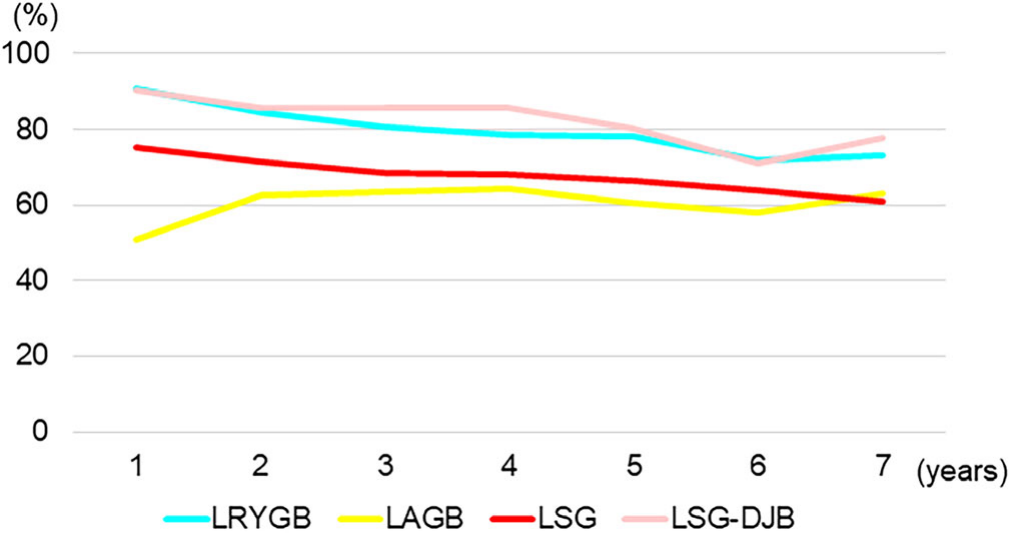
Percentage of excess weight loss after major bariatric/metabolic surgery by procedure. LAGB, laparoscopic adjustable gastric banding; LRYGB, laparoscopic Roux‐en‐Y gastric bypass; LSG, laparoscopic sleeve gastrectomy; LSG‐DJB, laparoscopic sleeve gastrectomy with duodenojejunal bypass

The mean preoperative weight and BMI were 116.4 kg and 43.0 kg/m^2^ in LRYGB, 109.2 kg and 39.7 kg/m^2^ in LAGB, 116.6 kg and 42.8 kg/m^2^ in LSG, and 110.2 kg and 40.1 kg/m^2^ in LSG‐DJB, respectively. The mean weight loss and %EWL after the major bariatric/metabolic procedures are shown in Table 2. These bariatric/metabolic procedures resulted in a mean weight loss of more than 25 kg and a mean %EWL of 60% at 7 years after the operation (Figure 3).

**TABLE 2:** Mean weight loss and percentage of excess weight loss (%EWL) after major bariatric/metabolic procedures

	Weight loss (kg)						
Procedure	1 y	2 y	3 y	4 y	5 y	6 y	7 y
LRYGB	40 (n = 206)	40 (n = 160)	40 (n = 150)	40 (n = 116)	39 (n = 84)	38 (n = 31)	39 (n = 22)
LAGB	22 (n = 47)	27 (n = 38)	26 (n = 38)	27 (n = 32)	26 (n = 32)	27 (n = 25)	27 (n = 19)
LSG	33 (n = 1877)	33 (n = 1118)	33 (n = 720)	33 (n = 496)	33 (n = 342)	33 (n = 86)	32 (n = 52)
LSG‐DJB	32 (n = 282)	32 (n = 227)	30 (n = 181)	31 (n = 125)	28 (n = 93)	29 (n = 78)	28 (n = 20)
				%EWL			
LRYGB	90 (n = 206)	84 (n = 160)	81 (n = 150)	79 (n = 116)	78 (n = 84)	72 (n = 31)	73 (n = 22)
LAGB	51 (n = 47)	63 (n = 38)	63 (n = 38)	64 (n = 32)	60 (n = 32)	58 (n = 25)	63 (n = 19)
LSG	75 (n = 1877)	72 (n = 1118)	68 (n = 720)	68 (n = 496)	66 (n = 342)	64 (n = 86)	61 (n = 52)
LSG‐DJB	90 (n = 282)	86 (n = 227)	86 (n = 181)	86 (n = 125)	80 (n = 93)	71 (n = 78)	78 (n = 20)

Abbreviations: LAGB, laparoscopic adjustable gastric banding; LRYGB, laparoscopic Roux‐en‐Y gastric bypass; LSG, laparoscopic sleeve gastrectomy; LSG‐DJB, laparoscopic sleeve gastrectomy with duodenojejunal bypass.

## 4 DISCUSSION

The first nationwide survey on laparoscopic bariatric surgery organized by JELTO (now JCOMS) showed that 340 laparoscopic bariatric procedures were performed in the 10 years between 2000 and 2009, including 70 in patients with obesity in 2009 alone.^1^ By the end of 2019, there had been more than a 10‐fold increase in the number of surgeries, with 757 patients undergoing a procedure in 2019. However, fewer procedures are performed in Japan than in the other Asia–Pacific countries. In 2018, representatives from 17 Asia–Pacific countries reported the current status of bariatric/metabolic surgery and related problems at the Asia‐Pacific Metabolic and Bariatric Surgery Society Congress in Tokyo.^5^ The summarized data for 2017 indicated that the countries with the fewest procedures per total population were Indonesia, Philippines and Japan. To reach the average number of procedures performed in the 17 countries represented, Japan would have to perform more than six times the number of procedures. In Japan in 2019, although 1.6‐fold more procedures were performed compared to in 2017, the number of procedures was still small relative to the total population.

Relatively few laparoscopic bariatric/metabolic surgeries are performed in Japan because the limited indications covered by National Health Insurance and a lack of awareness and comprehension by Japanese physicians and the general public. The Japanese guidelines announced by the Japan Society for Treatment of Obesity (JSTO) in 2013 stated that the indication for bariatric surgery was a BMI ≥ 35 kg/m^2^ and that for metabolic surgery was a BMI ≥ 32 kg/m^2^ in patients with diabetes or two other obesity‐related diseases. National Health Insurance in Japan has fully covered only LSG since 2014, but the indications have been limited to a BMI ≥ 35 kg/m^2^ and diabetes, hypertension, dyslipidemia, or obstructive sleep apnea syndrome. In addition, insurance has partially covered LSG‐DJB as an advanced medical treatment since 2018, but the indications have been limited to patients with a BMI ≥ 35 kg/m^2^ and uncontrolled diabetes. The frequency of obesity (BMI ≥ 30 kg/m^2^) in East Asian countries is lower than in other Asian countries, but the frequency of diabetes is comparable.^5^ Therefore, expanding the indications for metabolic surgery covered by National Health Insurance is very important to ensure that patients with obesity and diabetes in Japan receive better treatment.

At present, much evidence on the safety and effectiveness of bariatric/metabolic surgery has been published throughout the world, and the guidelines from the second Diabetes Surgery Summit have also been announced.^6^ However, Japanese physicians and the general public have not always been aware of this evidence. In addition, some Japanese physicians may think that bariatric/metabolic surgery is not needed for their patients with diabetes because recently developed antidiabetic drugs can reduce weight and control the diabetes condition well. Also, some Japanese physicians may misunderstand that surgical treatment is the final approach to diabetes treatment, particularly in elder patients for whom metabolic surgery is less effective because of a longer duration of diabetes treatment and insulin administration, as well as insufficient endogenous secretion of insulin.^7^, ^8^ This lack of understanding is not unique to Japan, as physicians and primary care providers in Western countries likewise do not always have detailed knowledge of bariatric/metabolic surgery.^9^, ^10^ Regardless, we will certainly continue to promote educational activities among Japanese physicians and the general public.

The sixth nationwide survey clearly showed an increase in the number of Japanese institutions that have introduced laparoscopic bariatric/metabolic surgery since 2017. In March 2018, JSTO and JSES cooperatively announced a position statement on the requirements for institutions planning to introduce laparoscopic bariatric/metabolic surgery.^11^ The statement outlined the following requirements: a surgeon who has been sufficiently trained under a supervisor skilled in laparoscopic bariatric/metabolic surgery; equipment and instruments for laparoscopic bariatric/metabolic surgery; a multidisciplinary team approach; standardized procedures and clinical pathways; periodic team conferences and support groups; participation in seminars organized by JSES and JSTO; registration of cases in the JSTO system; and acquisition of a center of excellence certification by JSTO. Furthermore, the statement offered guidance on avoiding accidents, complications, and insufficient weight loss due to inadequate preparation. Fortunately, there have been no reports of serious accidents in Japan since the statement's release.

In Japan, the incidence of gastric cancer has continuously decreased since the 1970s, but its mortality rate is still higher than in Western countries.^12^ This is why the excluded stomach after Roux‐en‐Y gastric bypass (RYGB), which cannot be checked by usual endoscopy, has been thought to be problematic in Japanese patients. A group at Chiba University started to perform open RYGB in 1982 but moved to vertical banded gastroplasty because of the problem of gastric cancer from 1984.^1^ Until now, 17 patients with gastric malignant tumors in the excluded stomach after RYGB have been reported worldwide.^13^ A recent review article reported that 6 of the 17 patients (35.3%) had tumors that were considered unresectable due to stage IV cancer, whereas 9 patients (52.9%) received subtotal gastrectomy with lymphadenectomy. The disease‐related mortality was 33.3% after gastrectomy (follow‐up duration, 3‐26 months), and their prognoses were poor. In May 2007, JSES released a position statement on patient selection and postoperative care for LRYGB.^14^ This statement explained the necessity of preoperative upper gastrointestinal endoscopy; the essentiality of a preoperative examination for *Helicobacter pylori* infection and atrophic gastritis; the difficulty of performing an upper gastrointestinal endoscopy on the bypassed stomach after LRYGB; and the need for close postoperative follow‐up of the bypassed stomach. Since then, LSG has rapidly increased, and insurance coverage has expanded to include this procedure. In addition, Kasama et al developed a new metabolic surgical procedure, LSG‐DJB, for Japanese patients with obesity and diabetes in 2007,^15^ and their recent paper reported excellent 5‐year results with regard to weight loss and diabetes remission.^16^

In clinical practice, GERD is often diagnosed by using questionnaires such as the Reflux Disease Questionnaire and Frequency Scale for the Symptoms of GERD.^17^, ^18^ However, these questionnaires have limitations when compared with upper gastrointestinal endoscopy and pH monitoring.^19^ The US guidelines strongly recommend that a presumptive diagnosis of GERD be established based on typical symptoms of heartburn and regurgitation and that empiric medical therapy with a PPI be administered at diagnosis.^20^ The PPI test has a limited ability to identify patients with GERD, but patients with reflux esophagitis had significant positive results compared to patients without GERD symptoms or with GERD but not esophagitis.^21^ Today, preoperative gastrointestinal endoscopy is routinely performed in all institutions in Japan, but postoperative endoscopy is not always performed. In addition, the questionnaires and pH monitoring used to diagnose GERD are not routinely performed before and after bariatric/metabolic surgery. Therefore, in this survey, postoperative GERD was defined as having GERD symptoms and requiring the administration of a PPI. In the previous five surveys, there was no definition of postoperative reflux esophagitis and GERD, and the incidence after LSG ranged from 1.5% to 7.9%. In this survey, the incidence of GERD after LSG was 12.1%, but it may have been underestimated because patients lacked GERD symptoms and were lost to follow‐up. In a previous study by Endo et al, postoperative endoscopy revealed reflux esophagitis in 23 of 54 patients (42.6%).^22^

In this study, the four major laparoscopic bariatric/metabolic procedures resulted in a mean weight loss of more than 25 kg and a mean %EWL of 60% at 7 years in Japanese patients with obesity. The famous systematic review by Buchwald et al found that weight loss and %EWL after RYGB were 44 kg and 62%, respectively, and those after LAGB were 29 kg and 48%, respectively.^23^ Another systematic review reported that %EWL ranged from 57% to 67% at 3 years after bariatric surgery, including LRYGB, LAGB, and LSG.^24^ The survey at the Fourth International Consensus Summit on Sleeve Gastrectomy reported a %EWL of about 50% at 6 years after LSG.^25^ Therefore, the weight loss results in Japan were comparable or superior to those in Western countries.

The first Japanese nationwide survey showed total morbidity rates of 12.2% for LRYGB, 10.9% for LAGB, and 7.8% for LSG.^1^ The morbidity rates for LRYGB and LSG in this study were much higher, 29.8% and 16.8%, respectively (Table 1). Since the second survey, the total morbidity rates for LRYGB have been higher than 25%, indicating that complications such as anastomotic stenosis and anastomotic ulcer might have been underestimated and/or not counted in the first survey. These two complication rates were much higher than those in the United States and may be related to initial immature techniques.^26^ With regard to LSG, this was the first survey to define GERD, and the resulting incidence rate was much higher than in previous reports. In this survey, the total morbidity rate, excluding GERD, was only 4.7%, which is much lower than in the first survey.

In conclusion, this nationwide survey conducted by JCOMS showed the current status and outcomes of laparoscopic bariatric/metabolic surgery in Japan and indicated that it was safely and effectively performed between 2000 and 2019.

## DISCLOSURE OF INTERESTS

The authors have no conflicts of interest to declare and received no financial support for this survey.

## AUTHOR CONTRIBUTIONS

All authors are in agreement with the content of this manuscript. Consistent with the latest guidelines of the International Committee of Medical Journal Editors, each author's contribution to the paper is to be quantified.

## ETHICS STATEMENT

All data collection and analyses were performed in accordance with the ethical standards of the Declaration of Helsinki, and the survey was approved by the Ethics Committee of Oita University Faculty of Medicine.
